# A Hint or Two

**Published:** 1894-01

**Authors:** C. Carleton Smith

**Affiliations:** Philadelphia, Pa.


### A HINT OR TWO.

C. CARLETON SMITH, M. D., PHILADELPHIA, PA.

When you have a case of colic with severe *burning* pains, do not fix your mind on Arsenicum to the exclusion of other remedies, but keep in mind Solanum-nig. The abdominal pains of this latter drug are very similar to Colocynth, also—*i. e.,* better from bending forward and from pressure. But unlike Colocynth the pains extend upward toward the heart and left shoulder.

Sinapis-nig. also has intense burning in stomach with colic, somewhat like Colocynth, with this difference, the pains come on while patient is bent forward, but are instantly relieved by sitting up straight.

When a patient complains of an attack of acute catarrh, and says that there is a profuse watery discharge from the right nostril while the left is completely stopped up, with chilly sensation alternating with heat, similar to Aconite, think of Solanum-nig.

In lumbago, extorting cries from the sufferer, compelling him to walk bent over, though the least movement causes intense agony, don't give Bryonia, but relieve the case with Solanum Tuberosum Ægrotans. Cold water under this drug causes shock either from washing the face or drinking.

For an excessively dry spasmodic cough, always coming on toward evening, preceded by tickling in trachea, think of Stillingia Sylvatica.

